# Evaluation of the appropriateness of pediatric seizure Management in Primary Care Clinics

**DOI:** 10.1016/j.ebr.2026.100891

**Published:** 2026-07-27

**Authors:** Shimpei Matsuda, Takato Akiba, Natsumi Kondo, Masayo Takegami, Takashi Tsukakoshi, Hiromichi Shoji

**Affiliations:** a Department of Pediatrics and Adolescent Medicine, Juntendo University Graduate School of Medicine, Tokyo, Japan; b Department of Pediatrics, Kamisu Saiseikai Hospital, Ibaraki, Japan; c NYS Medical Inc, Japan

**Keywords:** Pediatric seizures, Status epilepticus, Buccal midazolam, Primary care, Japan, Febrile seizures

## Abstract

**Background:**

Effective management of any pediatric seizure event — not only febrile seizures — is a core requirement of pediatric primary care. In Japan, where the background prevalence of febrile seizures (FSs) is high (8–10% of children), pediatric primary care clinics may encounter status epilepticus (SE) requiring immediate antiseizure medication. Buccal midazolam was approved in Japan in December 2020. The pediatric rescue medication landscape in Japan differs substantially from many other countries: rectal diazepam gel is not approved, and intranasal diazepam was not yet on the market during the study period. To our knowledge, primary-care-level data on the incidence and management of in-clinic pediatric seizures in Japan remain limited.

**Methods:**

This multicenter retrospective observational study included pediatric patients (0–18 years) who experienced seizures during clinic visits at 32 primary care clinics in Japan between November 2023 and October 2024. Clinical data were extracted from electronic medical records. SE was defined as a generalized tonic-clonic seizure lasting ≥5 min. Statistical analyses included chi-square tests to assess the relationships between seizure onset time of day, monthly occurrence, and emergency transport. Among transported patients, the appropriateness of buccal midazolam administration was evaluated against pre-specified criteria based on the Japanese package insert.

**Results:**

Of 967,417 eligible pediatric outpatient visits (after exclusion of 191,009 visits by patients aged >18 years), 132 (0.014%) involved an in-clinic seizure. Of these, 52 (39.4%) required emergency transport. No significant differences were observed in seizure occurrence across time of day (χ^2^(2, *n* = 132) = 0.32, *p* = 0.85) or months (χ^2^(11, n = 132) = 16.84, *p* = 0.11). Among the transported patients, 24 (46.2%) had SE (of whom 23 had febrile status epilepticus and 1 had an afebrile seizure following head trauma); seven (13.5%) had seizure clusters; the remainder did not meet criteria for SE, clusters, or focal seizures. Buccal midazolam was administered to 22 patients (42.3%) but was deemed appropriate for SE in only 14 (63.6%) of those administrations. Diazepam suppositories — a prophylactic, not a rescue, formulation in Japan — were administered in 19 (36.5%) cases, in some instances delaying buccal midazolam administration. All administered doses conformed to the age-stratified dosing schedule of the Japanese package inserts.

**Conclusions:**

Seizures in pediatric primary care occur sporadically and require continuous preparedness. Suboptimal management of SE, particularly delayed benzodiazepine administration and underutilization of buccal midazolam, was identified. Enhanced education focusing on SE recognition and appropriate medication use may improve the quality of seizure care in community pediatric settings.

## Highlights

• In-clinic seizures were rare: 132 of 967,417 pediatric primary care visits in Japan (0.014%).
• Transport per the Japanese package insert was required in 52 of 132 seizures (39.4%).
• Status epilepticus accounted for nearly half of seizure-related transports (24/52, 46.2%).
• Buccal midazolam was underused, while diazepam suppositories, a prophylactic agent in Japan, were often given.
• No seasonal or diurnal clustering was seen; training on SE and buccal midazolam is needed year-round.

## 1 Introduction

Effective management of seizures in pediatric primary care is crucial for ensuring patient safety and maintaining quality of care. Seizures are among the most common neurological emergencies in children and require a timely and appropriate response from healthcare professionals. This study addresses all in-clinic pediatric seizure events, regardless of etiology.

Febrile seizures (FSs) are the most common type of seizure in childhood. Although generally benign, the prevalence in Japan ranges from approximately 8% to 10%, which is notably higher than the 2%–5% observed in Western countries [1], [2], [3]. These epidemiological findings indicate that FS occurs relatively frequently in Japanese children, underscoring the importance of seizure preparedness and education in community pediatric practice. In the present cohort, FS likewise accounted for the majority of in-clinic seizure events; nonetheless, the analyses below address all seizure types, since clinic-level preparedness must encompass any pediatric seizure that may present.

Status epilepticus (SE) is operationally defined as a generalized tonic-clonic seizure lasting 5 min or longer (T1) and requires prompt administration of antiseizure medication (ASM) [4]. Approximately 1% of pediatric seizures are associated with serious underlying etiologies, such as acute encephalopathy, meningitis, or toxic metabolic disorders [5], [6]. Therefore, primary care physicians must be able to (1) identify SE and initiate treatment rapidly, (2) distinguish typical FSs from atypical presentations, and (3) recognize red flags that warrant urgent transfer to tertiary care facilities.

The pediatric rescue medication landscape in Japan differs substantially from those in many other countries, and this difference is essential context for the present analysis. The diazepam suppository is licensed in Japan for the prophylactic suppression of febrile seizure recurrence during a single febrile episode in children with a history of complex or recurrent febrile seizures, in line with the Japanese clinical practice guidelines for febrile seizures [38]. It requires approximately 15–30 min to reach therapeutic plasma concentrations and is therefore not pharmacokinetically suitable as a rescue agent for established SE. The fast-absorbed rectal diazepam gel formulation, which is widely used as out-of-hospital rescue therapy in the United States, is not approved in Japan. Intravenous (IV) midazolam is available and stocked at every participating clinic, but in pediatric primary care IV access is rarely established acutely, and the time required to obtain IV access can itself delay drug delivery. As a consequence, buccal midazolam — approved in Japan in December 2020 — is currently the only fast-onset, on-label, non-intravenous rescue therapy for pediatric SE in Japanese primary care.

Buccal midazolam has demonstrated high efficacy, rapid onset, and a favorable safety profile with minimal risk of respiratory depression [7], [8], [9], [10], [11]. However, the clinical effectiveness of buccal midazolam largely depends on clinicians' recognition of SE and their confidence in its administration. Inadequate training can lead to hesitation or delayed benzodiazepine use, prolonged seizures, and worsening outcomes [4]. Previous studies have shown that both handling errors and medication dosing errors are common in pediatric seizure management, highlighting the importance of structured education and practical training [12], [13].

This study aimed to investigate the incidence and clinical characteristics of seizure events occurring in pediatric primary care clinics; analyze emergency interventions, including ASM use and ambulance transfers; and identify key challenges in optimizing seizure management in community-based pediatric practice.

## 2 Methods

### 2.1 Study design and setting

This multicenter, retrospective observational study was conducted across 32 pediatric primary care clinics in Japan between November 1, 2023, and October 31, 2024. These clinics typically operate 365 days a year from 9:00 AM to 9:00 PM, providing continuous pediatric care. Each clinic was staffed by board-certified pediatricians or physicians with more than three years of outpatient experience. The study design adhered to the Strengthening the Reporting of Observational Studies in Epidemiology (STROBE) guideline [14].

All 32 participating clinics belong to a single pediatric primary care network and operate under a unified internal protocol for in-clinic seizure response. The standard rescue medication stock at every participating clinic comprises buccal midazolam, diazepam suppository, and intravenous midazolam. Intravenous diazepam, intravenous lorazepam, and other antiseizure medications are not stocked at any of the participating clinics, and no other antiseizure medications were used in the in-clinic management of seizures during the study period. The network covers a range of geographic settings (urban, suburban, and small-city) across multiple regions of Japan. In every region, secondary or tertiary pediatric care is reachable by ambulance within a clinically acceptable time frame, and all clinics have established arrangements for ambulance dispatch and inter-facility transfer.

### 2.2 Participants

Eligible participants were children aged 0–18 years who experienced seizures during clinic visits, as identified by attending physicians. Patients aged >18 years and those with incomplete or unavailable clinical data were excluded. Missing data were handled in accordance with standard statistical practices for retrospective studies [15]. Participants were categorized into two groups according to outcome: (1) those requiring emergency transportation, and (2) those discharged following observation and treatment.

### 2.3 Data collection

To ensure uniformity, clinical data were extracted from electronic medical records using a standardized collection form across all participating sites [16]. The following information was collected:

• Patient background: age, sex, date and time of seizure onset, and relevant medical history.
• Clinical characteristics: seizure semiology, fever, and duration of impaired consciousness.
• Treatment and outcomes: administration of buccal midazolam, diazepam suppository, intravenous midazolam, oxygen therapy, requirement for emergency transport, and documented reasons for transport decisions.

### 2.4 Operational definitions

In this study, status epilepticus (SE) refers specifically to convulsive SE, defined as a generalized tonic-clonic seizure of ≥5 min' duration, in line with the T1 threshold defined by the ILAE Task Force [4] and consistent with the Japanese guidelines for the treatment of pediatric status epilepticus [37].

FS was defined according to the American Academy of Pediatrics (AAP) guideline as seizures occurring in children aged 6 to 60 months in association with fever (≥38 °C), without evidence of central nervous system infection, metabolic disturbance, or prior afebrile seizures [19]. Simple FS was defined as a generalized seizure lasting less than 15 min that did not recur within 24 h, in accordance with the AAP guideline. However, because seizures lasting ≥5 min were classified as SE in this study (T1 threshold), the “simple FS” category effectively captured FS resolving in less than 5 min without recurrence. Seizures occurring in children older than 60 months in the presence of fever were classified separately as fever-associated seizures beyond the typical age range of FS.

The appropriateness of buccal midazolam use was evaluated against the following pre-specified criteria, derived from the Japanese package insert and the operational definition of SE above:

(i) Appropriate administration: administration to a patient with a generalized tonic-clonic seizure of ≥5 min' duration (i.e., once the operational definition of SE was established), at the age-stratified dose specified in the Japanese package insert.

(ii) Appropriate non-administration: cases in which buccal midazolam was withheld because the seizure resolved within 5 min; or the patient had repeated brief seizures with intervening recovery of consciousness during a single febrile episode; or the patient had a complex febrile seizure that did not progress to SE.

(iii) Inappropriate administration: (a) administration before the 5-min threshold for SE was reached; (b) repeat dosing of buccal midazolam, regardless of seizure recurrence, in keeping with Section 8.4.4 of the Japanese package insert (which states, in the authors' translation, “Do not administer an additional dose of this drug even if the seizure recurs after administration”); or (c) delayed administration due to prior administration of a non-rescue agent (i.e., diazepam suppository).

(iv) Inappropriate omission: failure to administer any antiseizure medication despite confirmed SE; or use of diazepam suppository as the principal seizure-terminating intervention in the setting of confirmed SE.

(v) Important clarification regarding delays. Delays in buccal midazolam administration were classified as inappropriate only when caused by prior administration of diazepam suppository (item (iii)(c) above). Delays attributable to obtaining intravenous access for IV midazolam were not classified as inappropriate, and patients in whom IV midazolam was administered were classified as appropriately managed regardless of the time required to obtain IV access.

#### 2.4.1 Transport guidance per Japanese package insert

Section 8.4.3 of the Japanese package insert for buccal midazolam stipulates that emergency medical transport be arranged after administration of the drug. In the authors' translation: “As a rule, after administration of this drug, arrange for emergency transport. If the seizure does not stop within 10 minutes, if the full dose could not be administered, or if shallow respiration or loss of consciousness is observed, the patient should be transported to a medical institution.” These protocols apply to all seizures meeting SE criteria, not only febrile seizures, and apply equally to patients managed by buccal midazolam or by other antiseizure medication. The Japanese guidelines for the treatment of pediatric status epilepticus [37] similarly recommend transport to a tertiary facility after administration of first-line antiseizure medication.

### 2.5 Statistical analysis

**Timing of seizures within clinics.** To determine whether seizure onset times were uniformly distributed during clinic operational hours (9:00–13:00, 15:00–18:00, and 18:00–21:00), a chi-square goodness-of-fit test was performed. The relationship between time of day and emergency transport was examined using the chi-square test of independence.

**Monthly trends.** To assess monthly variations (January–December) in seizure occurrence and emergency transport rates, a chi-square test of independence was conducted. Expected frequencies (≥5) were verified to meet test assumptions [17]. Bonferroni correction was applied when multiple comparisons were made [18].

**Seizure types and treatment interventions among emergency transport cases.** Among the seizure events requiring emergency transport, each event was first categorized by etiology as FSs, fever-associated seizures beyond the typical FS age range, or other causes, and was further classified according to its clinical characteristics as SE, seizure clusters, focal seizures, or seizures that did not meet these definitions. Treatment interventions were classified as buccal midazolam, diazepam suppository, intravenous midazolam, or no pharmacological treatment. The relationship between seizure type and intervention was analyzed using the chi-square test for independence.

All statistical analyses were performed using R version 4.3.0 (R Foundation for Statistical Computing, Vienna, Austria). Statistical significance was set at *p* < 0.05.

### 2.6 Ethical considerations

This retrospective study was approved by the Institutional Review Board of Juntendo University (approval no. E25-0180-H01). Because the study used existing clinical records obtained during routine clinical care and involved no additional intervention or biological sampling, the Institutional Review Board waived the requirement for individual written informed consent. In accordance with the Ethical Guidelines for Life Science and Medical Research Involving Human Subjects (Japan), information about the study was disclosed to the public and patients and their guardians were given the opportunity to opt out. Direct identifiers were removed prior to analysis. This study adhered to the Declaration of Helsinki (2013 revision) and complied with the STROBE reporting guidelines [14].

### 2.7 Data availability

Data from the study cohort (*n* = 132) are available upon reasonable request from the corresponding author. All direct identifiers were removed in accordance with the ICMJE Data Sharing Recommendations [20].

## 3 Results

**Fig. 1:**
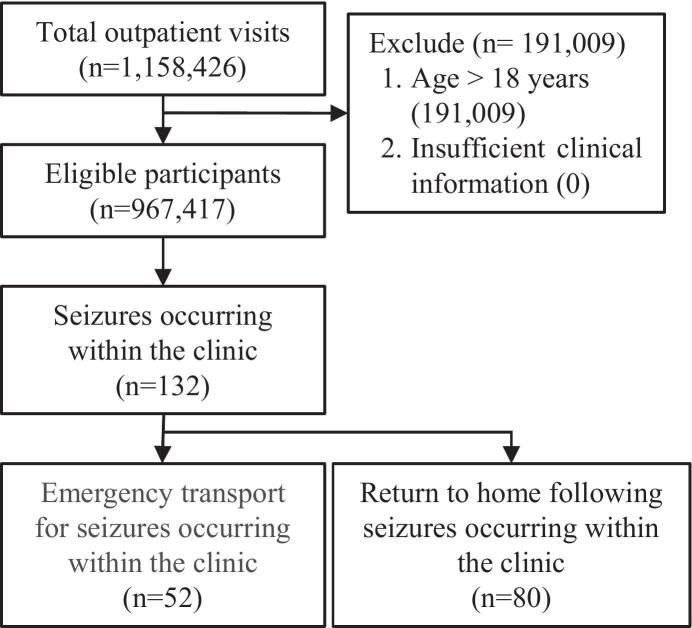
Study cohort and inclusion/exclusion criteria. Flow diagram showing the total number of pediatric patients screened, inclusion and exclusion criteria, and the final study population (*n* = 132) with seizures occurring within the clinic.

Of 967,417 eligible pediatric outpatient visits (after exclusion of 191,009 visits by patients aged >18 years; total clinic visits = 1,158,426), 132 (0.014%) involved an in-clinic seizure. Of these, 52 patients required emergency transport, and 80 were discharged (Fig. 1; Table 1). Diagnoses included one patient with an afebrile seizure following head trauma, one patient with epilepsy associated with tuberous sclerosis complex, 115 patients (87.1%) with FS, and 15 patients (11.4%) with fever-associated seizures (aged >5 years).

**Table 1:** Background information of eligible participants.

	**All seizures in clinic**	**Emergency transport**	**Discharged from clinic**
**Number of patients**	132	52	80
Male	73	27	46
Female	59	25	34
Median age (years)	2	2	2
Range (years)	0–12	0–12	0–10
**Time of day of seizure onset**			
9:00–13:00, n (%)	47 (35.6)	18 (34.6)	29 (36.3)
15:00–18:00, n (%)	42 (31.8)	13 (25.0)	29 (36.3)
18:00–21:00, n (%)	43 (32.6)	21 (40.4)	22 (27.5)
Blood glucose measurement, n	2	1	1

### 3.1 Timing of seizures occurring within clinics

**Fig. 2:**
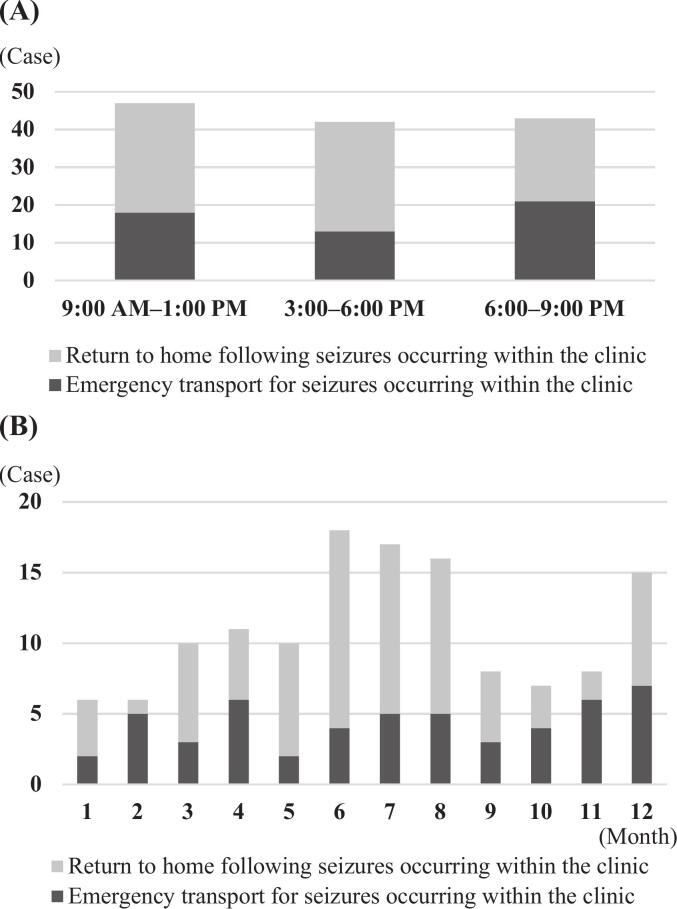
Timing and frequency of seizures occurring within the clinic. **(A)** Frequency of seizures occurring during three time-of-day windows (9:00–13:00, 15:00–18:00, and 18:00–21:00). Cases are categorized as those requiring emergency transport or those discharged home. **(B)** Monthly frequency of seizure onset and proportion of emergency transport cases, categorized as “Emergency transport for seizures within the clinic” and “Return home after seizures within the clinic.”

The observed timing of seizure onset was 47 from 9:00–13:00, 42 from 15:00–18:00, and 43 from 18:00–21:00. The chi-square test showed no significant deviation from uniform distribution (χ^2^(2, *n* = 132) = 0.32, *p* = 0.85). Similarly, no significant association was observed between seizure onset time of day and emergency transport (χ^2^(2, *n* = 132) = 2.46, *p* = 0.29) (Fig. 2A; Table 2).

**Table 2:** Observed and expected frequencies of emergency transport by time of day.

Time of day	Observed (No transport)	Observed (Transport)	Expected (No transport)	Expected (Transport)
9:00–13:00	30	17	28.5	18.5
15:00–18:00	28	14	25.5	16.5
18:00–21:00	22	21	26.1	16.9

### 3.2 Monthly trends in seizure onset and emergency transport

No significant association was found between the month of seizure onset and emergency transport (χ^2^(11, n = 132) = 16.84, *p* = 0.11). The number of patients requiring emergency transport ranged from 2 in January to 7 in December (Fig. 2B; Table 3). Expected frequencies were ≥ 5 for all categories, confirming the adequacy of sample size. These findings suggest that seizure occurrence and emergency transportation were evenly distributed throughout the year.

**Table 3:** Observed and expected monthly frequencies of seizure onset and emergency transport.

Month	Emergency transport	Observed frequency	Expected frequency
January	(−)	4	3.6
	(+)	2	2.4
February	(−)	1	3.6
	(+)	5	2.4
March	(−)	7	6.1
	(+)	3	3.9
April	(−)	5	6.7
	(+)	6	4.3
May	(−)	8	6.1
	(+)	2	3.9
June	(−)	14	10.9
	(+)	4	7.1
July	(−)	12	10.3
	(+)	5	6.7
August	(−)	11	9.7
	(+)	5	6.3
September	(−)	5	4.8
	(+)	3	3.2
October	(−)	3	4.2
	(+)	4	2.8
November	(−)	2	4.8
	(+)	6	3.2
December	(−)	8	9.1
	(+)	7	5.9

### 3.3 Seizure types and treatment interventions in patients requiring emergency transport

Among the 52 patients requiring emergency transport, seizure types included SE in 24 patients (46.2%) — of whom 23 had febrile status epilepticus and 1 had an afebrile seizure following head trauma — seizure clusters in 7 (13.5%), focal seizures in 2 (3.8%), and 19 patients (36.5%) did not fulfill the criteria for SE, seizure clusters, or focal seizures.

**Fig. 3:**
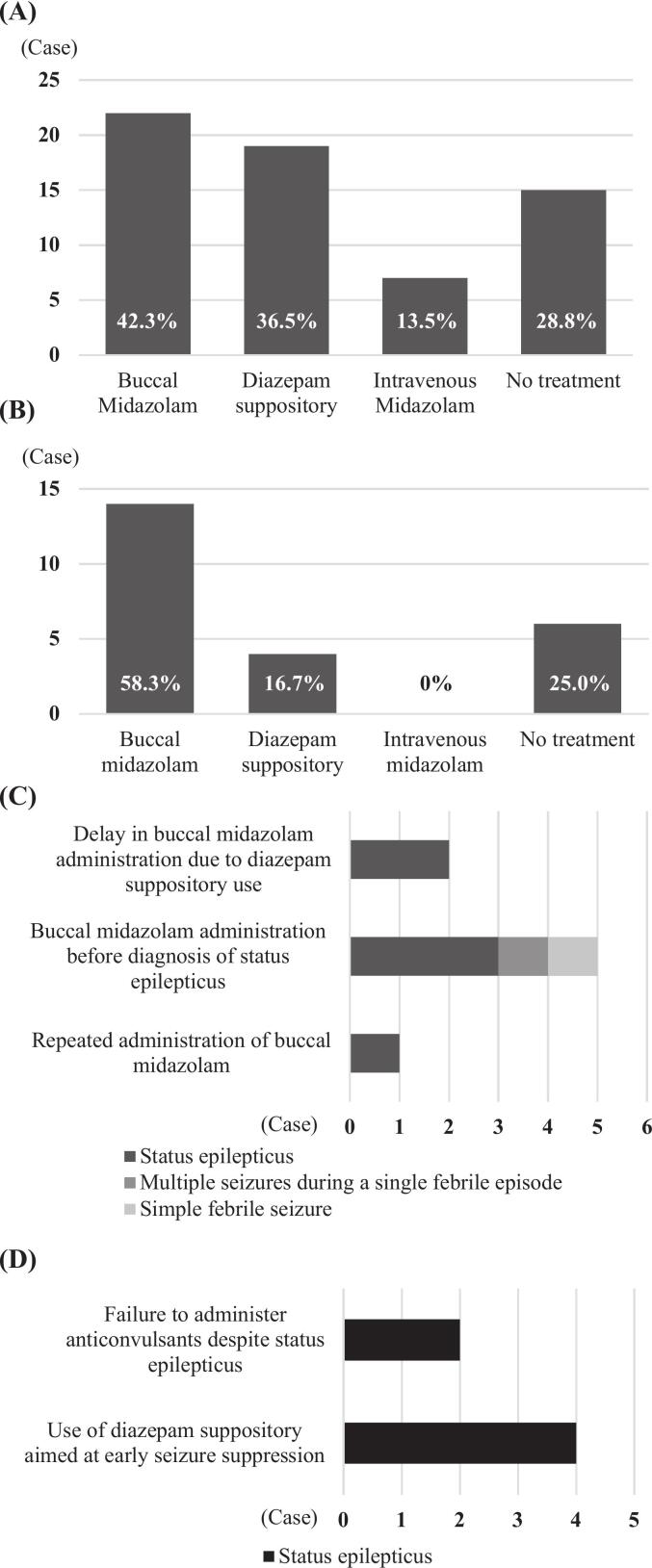
Seizure types and treatment interventions in emergency transport cases. **(A)** Distribution of treatment interventions among 52 cases requiring emergency transport. Interventions are categorized into four groups: buccal midazolam, diazepam suppository, intravenous midazolam, and no pharmacological treatment. **(B)** Distribution of treatment interventions among 24 cases of SE. **(C)** Analysis of eight cases of non-adherent use of buccal midazolam. The main reasons for use not consistent with indication criteria were: delayed administration due to prior diazepam suppository use, administration before confirmation of SE, and repeated dosing of buccal midazolam. Cases are further categorized into four seizure types: multiple seizures during a single febrile episode, SE, focal seizure, and other/unspecified seizures. **(D)** Analysis of six cases in which omission of buccal midazolam was judged as non-adherent to indication criteria. The main reasons were early administration of diazepam suppositories for seizure suppression and failure to administer antiseizure medication despite confirmed SE.

The treatment interventions were as follows: buccal midazolam, 22 patients (42.3%); diazepam suppository, 19 (36.5%); intravenous midazolam, seven (13.5%); and no pharmacological treatment, 15 (28.8%) (Table 4; Fig. 3A–B). All administered doses of buccal midazolam, diazepam suppository, and intravenous midazolam in this cohort conformed to the age-stratified dosing schedule specified in the Japanese package inserts; no instance of an administered dose falling outside the on-label dose range was identified. None of the patients had been instructed to use or were prescribed buccal midazolam for seizure or SE management before their clinic visit.

**Table 4:** Medications used during emergency transport for seizures occurring in the clinic.

Total transported	52 patients	
Buccal midazolam	22 patients	42.3%
Diazepam suppository	19 patients	36.5%
Intravenous midazolam	7 patients	13.5%
No treatment	15 patients	28.8%

Among the patients with SE (*n* = 24), buccal midazolam was administered in 14 (58.3%), diazepam suppository in four (16.7%), and no medication was administered in six (25%). Of the 22 patients who received buccal midazolam, 14 (63.6%) were treated appropriately for SE, while 8 administrations (36.4%) were considered inappropriate. The reasons for non-adherence included prior administration of a diazepam suppository causing a delay (*n* = 2), administration before confirming SE (*n* = 5), and repeated buccal midazolam dosing (*n* = 1) (Table 5; Table 6; Fig. 3C).

**Table 5:** Appropriate use of buccal midazolam in emergency transport for in-clinic seizures.

**Use of buccal midazolam**	**22 patients**	
Of which: appropriately deemed necessary	14 patients	63.6%
**Non-use of buccal midazolam**	**30 patients**	
Of which: appropriately deemed unnecessary	24 patients	80.0%

**Table 6:** Patients with inappropriate use of buccal midazolam.

Reason	Total (n)	Multiple seizures during a single febrile episode	SE	Focal seizure	Other / unspecified
Total inappropriate (patients)	8	1	6	0	1
Reason 1: Delay due to prior diazepam suppository	2 (25.0%)	0	2 (33%)	0	0
Reason 2: Administration before SE confirmation	5 (62.5%)	1 (100%)	3 (50%)	0	1 (100%)
Reason 3: Repeat administration of buccal midazolam	1 (12.5%)	0	1 (16.7%)	0	0

Conversely, of the 30 patients who did not receive buccal midazolam, 24 (80%) were appropriately managed (i.e., did not meet the operational definition of SE at the time of clinical assessment, in keeping with the criteria specified in Methods 2.4). However, six patients (20%) were classified as inappropriate due to early diazepam suppository use (*n* = 4) or omission of ASM despite confirmed SE (n = 2) (Table 7; Fig. 3D).

**Table 7:** Patients with inappropriate omission of buccal midazolam.

Reason	Total (n)	Multiple seizures during a single febrile episode	SE	Focal seizure	Other / unspecified
Total inappropriate omission (patients)	6	0	6	0	0
Reason 1: Diazepam suppository used for early seizure suppression	4 (66.7%)	0	4 (66.7%)	0	0
Reason 2: Failure to administer ASM despite confirmed SE	2 (33.3%)	0	2 (33.3%)	0	0

## 4 Discussion

This multicenter study examined the frequency, timing, and management of seizures in pediatric primary care clinics in Japan. The annual incidence of in-clinic seizures was 0.014% (132/967,417 eligible visits), and no significant variation was observed across time of day or months, suggesting that seizure events can occur unpredictably throughout the day and year. This underscores the importance of continuous readiness among primary care physicians to effectively manage seizures.

### 4.1 Clinic setting and generalizability

All 32 participating clinics belong to a single pediatric primary care network and operate under a unified internal protocol. Every clinic stocks buccal midazolam, diazepam suppository, and intravenous midazolam as standard rescue medications; intravenous diazepam, intravenous lorazepam, and other ASMs are not stocked or used. The clinics are distributed across urban, suburban, and small-city settings in multiple regions of Japan, and in every region secondary or tertiary pediatric care is reachable by ambulance within a clinically acceptable time frame. This institutional uniformity is a strength of the present study, in that it isolates clinical decision-making from variation in drug availability. However, it limits the direct generalizability of our findings to settings (such as many primary care clinics in the United States) in which on-site rescue medications and rapid ambulance transfer are not standard.

### 4.2 Seizure incidence in the context of japanese primary care

According to a 2021 report by the Welfare and Medical Service Agency, Japanese pediatric outpatient clinics operate on average 256 days per year, treating approximately 42.7 patients daily, which is equivalent to approximately 10,931 visits annually [21]. Extrapolating from our data, a typical pediatric clinic may encounter approximately 1.5 patients with in-clinic seizures per year, of whom 0.6 require emergency transport. Although relatively uncommon compared with other pediatric emergencies, seizures may still occur during clinic visits, and a subset can progress to severe outcomes requiring rapid and standardized management.

### 4.3 Suboptimal management of status epilepticus

A key finding of this study was the suboptimal administration of ASM for some patients with SE; in particular, the inappropriate use of diazepam suppositories for ongoing seizures was notable. As outlined in the Introduction, the diazepam suppository in Japan is licensed for the prophylactic suppression of febrile seizure recurrence within a single febrile episode and is not pharmacokinetically suitable as a rescue agent for established SE; the fast-absorbed rectal diazepam gel formulation widely used in the United States is not approved in Japan. Prolonged convulsive seizures lead to time-dependent internalization of synaptic GABA-A receptors and a corresponding decline in the responsiveness of any benzodiazepine [22], [23]; this phenomenon is a property of all benzodiazepines rather than of any specific formulation, and it underscores the importance of timely first-line treatment. Challenges in early management of seizures have been extensively debated in international studies. In the multicenter ESETT trial, more than 75% of patients received subtherapeutic initial doses of benzodiazepines, reflecting a pervasive underdosing pattern in emergency SE management [24].

In Japan, the first-line treatment for SE in primary care is buccal midazolam, with intravenous midazolam available where IV access can be obtained. Buccal midazolam, approved in 2020, is a simple, effective, and safe rescue medication for pediatric SE [9], [11]. Although it has been recommended as a first-line rescue therapy in international guidelines [25], [26], its clinical use remains limited [11], [27]. This is likely due to physicians' unfamiliarity with the medication and concerns regarding respiratory depression. However, a recent multicenter retrospective study in Japan demonstrated that buccal midazolam achieved seizure cessation within 10 min in 43% of episodes, with no patients requiring ventilatory support, even in tertiary care settings [11]. These findings support its safety and highlight the importance of early administration, ideally within 15 min of seizure onset. Targeted educational initiatives and simulation-based training could help improve clinicians' confidence and reduce delays in seizure treatment [12], [13].

It is important to note that the Japanese package-insert restriction on repeat dosing of buccal midazolam (Section 8.4.4) pertains specifically to the same product and route, and is conceptually distinct from international guideline recommendations to administer a second-line benzodiazepine via an alternative route (e.g., intravenous) when seizures persist. The repeat-dosing case classified as inappropriate in the present cohort was so classified solely on the basis of this product- and route-specific package-insert provision, and not because the international principle of escalating to a second benzodiazepine when seizures persist is itself inappropriate.

Conversely, some patients received ASMs before SE was confirmed, with seizures lasting less than five minutes. In febrile seizures, which accounted for the majority of seizure events in this study, approximately 75–83% of episodes spontaneously terminate within five minutes [28], [29]. For children with a known history of prolonged seizures or epilepsy who have an established rescue plan, early caregiver administration of buccal or intranasal midazolam around the 5-min threshold is consistent with current international recommendations [4], [26], [30]. In primary care encounters without such a prior plan, observation and preparation for treatment within approximately five minutes are considered appropriate. Excessive or repeated benzodiazepine dosing can increase the risk of respiratory depression, although large randomized and guideline-informed studies have reported that clinically significant respiratory events are uncommon when recommended doses and intervals are followed [31], [32]. Therefore, optimal pediatric seizure management should prioritize the timely administration of appropriate doses while maintaining safety, supported by continuous education and training of healthcare providers.

### 4.4 Decision to transport and protocol coupling with buccal midazolam administration

As described in Methods 2.4.1, administration of buccal midazolam and the request for emergency transport are protocol-coupled by the Japanese package insert. Consistent with this, every patient who received buccal midazolam in our cohort was transported, and no discharged patient received buccal midazolam. Within this protocol, the decision to transport is driven by the clinical characteristics of the seizure itself — complex febrile seizure features, repeated seizures within a single febrile episode, or SE — rather than by whether or not a rescue medication has been given. Brief seizures with preserved consciousness in patients meeting criteria for simple FS were generally managed by observation. Our data are therefore not suitable for evaluating whether earlier or different rescue medication administration would have changed the decision to transport.

### 4.5 Country-specific differences in pediatric rescue medication practice

Internationally, the landscape of pediatric out-of-hospital seizure rescue medications differs substantially between regions. In the United States, intranasal midazolam and intranasal diazepam have become first-choice options, in part because of concerns about aspiration during oral administration in an actively convulsing child; rectal diazepam gel also remains widely used [33], [34]. In Japan, by contrast, rectal diazepam gel is not approved, and intranasal diazepam was approved in Japan in June 2025 and launched in December 2025, that is, after the conclusion of our study period; it is therefore not represented in our cohort. Although intranasal diazepam represents a potential future option in Japanese pediatric clinical practice, its current Japanese label restricts use to children aged ≥2 years. Given that 42.9% of patients in our cohort were under 2 years of age — the age band in which febrile seizures are most prevalent — the applicability of intranasal diazepam in pediatric primary care under the present labeling remains constrained. The current Japanese clinical practice guidelines for the treatment of pediatric status epilepticus [37] do not yet provide specific recommendations for intranasal diazepam, since domestic experience is still accruing. Within these constraints, buccal midazolam is currently the most rapidly deliverable on-label rescue therapy for pediatric SE in Japanese primary care, and our findings should be interpreted within this country-specific context rather than generalized to other settings.

### 4.6 Limitations

This study had several limitations.

First, the data on outcomes during and after emergency transport were incomplete. As a result, it was not possible to determine whether respiratory depression or other adverse events occurred following antiseizure medication administration, or to assess the influence of initial management on clinical prognosis. Furthermore, we did not have access to receiving-hospital records, and the appropriateness of each individual transport decision could therefore not be independently adjudicated.

Second, although the dataset records the fact of medication administration, the precise minute of administration was not consistently documented in the free-text fields of the electronic medical records. Among the 52 transported patients, the time of seizure onset was documented in only 4 cases and the time of buccal midazolam administration was documented in only 4 cases. A quantitative analysis of seizure-onset-to-medication intervals was therefore not feasible. A prospective study with prespecified time-stamping is planned as the next phase of this research program.

Third, although all administered doses conformed to the age-stratified dosing schedule of the Japanese package inserts, we did not analyze potential mismatch between the standard age-based dose and individual body size (i.e., relative under- or over-dosing in children whose weight is markedly atypical for their age band), as the present dataset did not consistently capture body weight at the time of seizure.

Fourth, a definitive differentiation between epileptic seizures and paroxysmal nonepileptic events (PNEEs) was not possible. We use “PNEEs” in line with the ILAE-aligned terminology used in recent pediatric epilepsy literature [35]; this is a broader concept than psychogenic non-epileptic seizures and we do not assume a psychogenic origin for any event in this cohort. In primary care, where video documentation and routine electroencephalographic assessment are not feasible, we cannot fully exclude the possibility that a small subset of prolonged motor events were PNEEs that clinically resembled SE [36]. Greater awareness and continued educational initiatives among pediatric primary care physicians may help improve the recognition and management of these events in the future.

Finally, the dataset was limited to participating clinics, and the findings may not be generalizable to all Japanese pediatric primary care settings. Future prospective studies that incorporate longitudinal follow-up and interventional educational programs are warranted to validate and expand upon these findings.

## 5 Conclusion

This multicenter study delineates the current landscape of pediatric seizure management in Japanese primary care clinics. Although seizures occur sporadically throughout the day, maintaining continuous preparedness among healthcare providers is essential. This study identified suboptimal management of SE, particularly the inappropriate use of diazepam suppositories and underutilization of buccal midazolam. Strengthening educational initiatives that emphasize the safety and appropriate use of buccal midazolam and the early recognition of SE are necessary to improve the quality and timeliness of acute seizure care in community pediatric settings.

## Ethical Considerations

This retrospective study was approved by the Institutional Review Board of Juntendo University (approval no. E25-0180-H01). Because the study used existing clinical records obtained during routine clinical care and involved no additional intervention or biological sampling, the Institutional Review Board waived the requirement for individual written informed consent. In accordance with the Ethical Guidelines for Life Science and Medical Research Involving Human Subjects (Japan), information about the study was disclosed to the public and patients and their guardians were given the opportunity to opt out. Direct identifiers were removed prior to analysis. This study adhered to the Declaration of Helsinki (2013 revision) and complied with the STROBE reporting guidelines.

## Funding

This research did not receive any specific grant from funding agencies in the public, commercial, or not-for-profit sectors.

## Author Contribution

Dr. Shimpei Matsuda conceptualized and designed the study, designed the data collection instruments, collected data, carried out the initial analyses, drafted the initial manuscript, and critically reviewed and revised the manuscript.

Dr. Takato Akiba substantially contributed to the study design, data interpretation, and methodological framework, and critically reviewed and revised the manuscript.

Natsumi Kondo and Masayo Takegami collected data and carried out the initial analyses.

Dr. Takashi Tsukakoshi and Dr. Hiromichi Shoji critically reviewed and revised the manuscript.

All authors have approved the final manuscript as submitted and agreed to be accountable for all aspects of this work.

## CRediT authorship contribution statement

**Shimpei Matsuda:** Writing – original draft, Visualization, Project administration, Methodology, Investigation, Formal analysis, Data curation, Conceptualization. **Takato Akiba:** Writing – review & editing. **Natsumi Kondo:** Resources. **Masayo Takegami:** Resources. **Takashi Tsukakoshi:** Writing – review & editing, Resources. **Hiromichi Shoji:** Writing – review & editing, Supervision.

## Declaration of competing interest

Shimpei Matsuda received consulting fees from CAPS Inc. under a service contract, and the contracted work included research-related tasks. This study was conducted at clinics operated by NYS Medical Inc. The other authors declare no conflicts of interest.

## Data Availability

The data will be available upon on request.
